# Hollow-polydopamine-nanocarrier-based near-infrared-light/pH-responsive drug delivery system for diffuse alveolar hemorrhage treatment

**DOI:** 10.3389/fchem.2023.1222107

**Published:** 2023-06-15

**Authors:** Lingyan Zhang, Mifang Li, Yeying Wang, Yibiao Liu, Feiyuan Zhang, Zhihao Lin, Yuling Zhang, Mingliang Ma, Shouju Wang

**Affiliations:** ^1^ Lab of Molecular Imaging and Medical Intelligence, Department of Radiology, Longgang Central Hospital of Shenzhen, Shenzhen, China; ^2^ Medical Frontier Innovation Research Center, The First Hospital of Lanzhou University, Lanzhou, China; ^3^ Shanghai Engineering Research Center of Molecular Therapeutics and New Drug Development, School of Chemistry and Molecular Engineering, East China Normal University, Shanghai, China; ^4^ Wenzhou Key Laboratory of Biophysics, Wenzhou Institute, University of Chinese Academy of Sciences, Wenzhou, China; ^5^ Lab of Molecular Imaging, Department of Radiology, The First Affiliated Hospital of Nanjing Medical University, Nanjing, China

**Keywords:** diffuse alveolar hemorrhage, hollow polydopamine, photothermal conversion, collaborative treatment, nanodrug loading platform

## Abstract

**Introduction:** Diffuse alveolar hemorrhage (DAH) is a serious complication caused by systemic lupus erythematosus (SLE). Tissue damage and changes in immune response are all associated with excessive free radical production. Therefore, removing excess reactive oxygen species are considered a feasible scheme for diffuse alveolar hemorrhage treatment. Cyclophosphamide is often used as the main therapeutic drug in clinics. However, CTX carries a high risk of dose-increasing toxicity, treatment intolerance, and high recurrence rate. The combination of therapeutic drugs and functional nanocarriers may provide an effective solution. PDA is rich in phenolic groups, which can remove the reactive oxygen species generated in inflammatory reactions, and can serve as excellent free radical scavengers.

**Methods:** We developed a hollow polydopamine (HPDA) nanocarrier loaded with CTX by ionization to prepare the novel nanoplatform, CTX@HPDA, for DAH treatment. The monodisperse silica nanoparticles were acquired by reference to the typical Stober method. PDA was coated on the surface of SiO_2_ by oxidation self-polymerization method to obtain SiO_2_@PDA NPs. Then, HPDA NPs were obtained by HF etching. Then HPDA was loaded with CTX by ionization to prepare CTX@HPDA. Then we tested the photothermal effect, animal model therapeutics effect, and biosafety of CTX@HPDA.

**Results:** Material tests showed that the CTX@ HPDA nanoplatform had a uniform diameter and could release CTX in acidic environments. The vitro experiments demonstrated that CTX@HPDA has good photothermal conversion ability and photothermal stability. Animal experiments demonstrated that the CTX@HPDA nanoplatform had good biocompatibility. The nanoplatform can dissociate in acidic SLE environment and trigger CTX release through photothermal conversion. Combining HPDA, which scavenges oxygen free radicals, and CTX, which has immunosuppressive effect, can treat pulmonary hemorrhage in SLE. Micro-CT can be used to continuously analyze DAH severity and lung changes in mice after treatment. The pulmonary exudation in the various treatment groups improved to varying degrees.

**Discussion:** In this study, we report a photothermal/PH-triggered nanocarrier (CTX@HPDA) for the precise treatment of SLE-DAH. CTX@HPDA is a simple and efficient nanocarrier system for DAH therapy. This work provides valuable insights into SLE treatment.

## 1 Introduction

Systemic lupus erythematosus (SLE) is a chronic autoimmune disease characterized by the presence of autoantibodies, which can lead to the formation of immune complexes and inflammatory responses in various organs, seriously threatening human health. Diffuse alveolar hemorrhage (DAH) is a serious complication of SLE, which has a dangerous onset, poor prognosis, and high mortality (Hannah and D'Cruz, 2019). The incidence of DAH in patients with SLE is only 0.6%–5.4% (Martínez-Martínez et al., 2017), but its mortality rate can be as high as 29.2%–61.9% (Jiang et al., 2021). This outcome may be related to the deposition of immune complexes in the alveolar wall, leading to cell necrosis and causing microvasculitis, especially capillaritis (Jarrot et al., 2019). Oxidative stress can promote SLE development by regulating the transduction of intracellular signal channels, the synthesis and degradation of extracellular matrix, the permeability of biofilm, and cell aging and apoptosis (Zhuang et al., 2022). Reactive oxygen species (ROS) is the direct cause of SLE. Tissue damage, reduction of T cell subsets, and changes in immune response are all associated with excessive free radical production (Bermejo Vicedo and Hidalgo Correas, 1997). Oxygen free radicalsis increased and antioxidant system is inhibited in SLE. Therefore, maintaining the balance of oxidative and antioxidant effects *in vivo* and removing excess ROS are considered a feasible scheme for DAH treatment. In addition, SLE occurs in an acidic environment because of a dramatic increase in metabolic activity and a decrease in intracellular glutathione (Pravda, 2019; Pravda, 2020).

At present, the main treatments for DAH in clinical practice are glucocorticoids and immunosuppressants, such as cyclophosphamide (CTX) (Al-Adhoubi and Bystrom, 2020). CTX has been approved by the FDA for SLE treatment. CTX can inhibit cell proliferation and has non-specific killing effect on B cells and T cells stimulated by antigen when they enter division, which can inhibit humoral and cellular immunity and reduce the production of autoantibodies (Ponticelli et al., 2018). However, the use of CTX alone carries a high risk of dose-increasing toxicity, myelosuppression, and high recurrence rate, without well defined efficacy in SLE-DAH yet owing to its adverse pharmacokinetics and biodistribution (Andrade et al., 2016; Guo et al., 2021). An approach to overcome this obstacle is to replace free CTX with CTX loaded into a drug delivery system, such as liposomes, polymers, and micelles (Quan et al., 2014). Nanocarriers controlling the release of the loaded drug is a promising therapeutic approach. Nanocarriers improve drug accumulation and release in pathological sites, improves the therapeutic effect in general, reduces the incidence and intensity of side effects, and can well achieve the purpose of treatment (Xie et al., 2020; Zhang et al., 2020). Polydopamine (PDA) is a biocompatible and biodegradable polymer with similar structure and chemical properties to melanin. PDA nanoparticles (NPs) can be used for drug delivery and photothermal therapy owing to their extensive molecular adsorption capacity, easy preparation, and high near-infrared (NIR) absorption (Ryu et al., 2018; Zhao et al., 2018; Ding et al., 2020; Wang et al., 2020; Li et al., 2021). In addition, PDA is rich in phenolic groups, which can remove the ROS generated in inflammatory reactions, and can serve as excellent free radical scavengers (Bao et al., 2018; Ma et al., 2022a; Chen et al., 2022). Zhao et al. (2018) used PDA nanoparticles to reduce ROS levels in murine macrophages. The anti-inflammatory ability of PDA nanoparticles was further demonstrated in mouse models of acute peritonitis and acute lung injury. PDA can reduce the production of proinflammatory cytokines and reduce neutrophil infiltration, thusing lung tissue damage. Moreover, PDA depolymerization under acidic conditions can enhance the release of loaded drugs (Cheng et al., 2017). However, pure PDA NPs generally do not have pores, which makes drug loading for tumor treatment difficult (Ao et al., 2018; Wu et al., 2022). We hypothesized that the hollow structure of hollow polydopamine (HPDA) NPs with high porosity and specific surface area can remarkably improve the reaction efficiency and drug loading capacity (Wang et al., 2021; Ma et al., 2022b).

In this work, we prepared a theragnostic nanoplatform based on HPDA coated with CTX, an immunosuppressant, for DAH-response drug delivery. HPDA, as a drug-carrying system, can carry CTX to inflammatory tissues and release CTX through photothermal effect under NIR light and pH response. The phenolic group of HPDA can scavenge oxygen radicals. This work aimed to obtain insights into DAH diagnosis and therapy.

## 2 Experimental section

### 2.1 Materials

Ammonium hydroxide (NH_3_·H_2_O, 25%–28%, w/w), tetraethyl orthosilicate (TEOS), dopamine (DA), hydrofluoric acid (HF), Tris (hydroxymethyl) aminomethane, and phosphate buffered saline (PBS) were obtained from Sigma Aldrich (Shanghai, China). Sodium hydroxide (NaOH) and anhydrous ethanol were purchased from Sinopharm Chemical Reagent Co., Ltd., (Shanghai, China). CTX was obtained from Macklin (Shanghai, China). Phorbol 12-myristate-13-acetate was purchased from Beyotime (Wuhan, China). All the chemical reagents were used directly as received. Milli-Q water purification system (Bedford, MA) was adopted to acquire deionized water.

### 2.2 Preparation of HPDA NPs

The monodisperse silica nanoparticles were acquired by reference to the typical Stober method (Flachsbart and Stöber, 1969). 80 mL absolute ethanol and 3.5 mL NH_3_·H_2_O were added to a certain amount of deionized water and stirred at 35°C for 30 min. Then 3.5 mL TEOS was dropped and stirred for 2 h to obtain SiO_2_ nanoparticles. The resulting SiO_2_ was dispersed in 40 mL Tris buffer (pH = 8.5). Tris buffer was prepared by Tris (hydroxymethyl) aminomethane and deionized water. Then 3.5 mL of dopamine hydrochloride solution (DA, 75 mg/mL) was quickly added and stirred for 12 h to obtain SiO_2_@PDA nanoparticles. The obtained SiO_2_@PDA nanoparticles were dispersed again in 40 mL of deionized water and absolute ethanol. An additional 5 mL HF solution was added and stirred overnight. Hollow PDA nanoparticles (HPDA) were obtained by centrifugation and washing with deionized water.

### 2.3 CTX loading and release

CTX was loaded into the HPDA NPs by mixing 2.5 mL of freshly prepared CTX solution (10 mg·mL^−1^) with 25 mL of HPDA NPs (1 mg·mL^−1^). The mixed solution was stirred in the dark for 36 h at 37°C. Then, the remaining CTX was wiped off by centrifugation. The loading capacity of CTX was calculated by the absorbance at 207 nm following the equation:

$$Loading\;capacity=\left(\frac{weight\;of\;loaded\;CTX}{weight\;of\;nanoparticles\;and\;loaded\;CTX}\right)\times 100\%$$ (1)

The CTX released from the HPDA NPs was obtained as follows. CTX@HPDA NPs (3 mg) were dispersed in 6.0 mL PBS solutions at pH 6.5 and 7.4, respectively. The solutions were stirred in a shaker at 37°C and centrifuged at certain time-points. The supernatants were collected, and the CTX@HPDA at the bottom was redispersed in fresh PBS solutions. The amounts of released CTX in the corresponding PBS supernatants at different times were obtained via the characteristic absorption of CTX.

### 2.4 General characterization

The morphology of the NPs were detected by transmission electron microscopy (TEM) at a voltage of 200 kV (JEM-2100, JEOL, Japan). The size and zeta potential of the NPs were recorded with a dynamic light scattering (DLS) system (Mastersizer 3000, Malvern Instrument, United Kingdom). Fourier transform infrared spectroscopy (FTIR) spectra were acquired by an INVENIO spectrometer (Bruker, GER). UV-Vis-NIR absorption spectra were measured on a UV-3600Plus spectrophotometer (Shimadzu, CN). Pathological images were obtained with an upright metallurgical microscope (Carl Zeiss, Axio Imager A2, Jena, Germany).

### 2.5 Photothermal effect of NPs

A NIR light source with a wavelength of 808 nm and a power density of 1.0 W·cm^−2^ was used to vertically irradiate a sample containing 200 μL of CTX@HPDA with different concentrations (0, 50, 100, 150, and 200 μg·mL^−1^) to test the photothermal performance of CTX@HPDA. Moreover, the temperature of 200 μg·mL^−1^ CTX@HPDA was recorded by adjusting the power density of the NIR laser to 0.5, 0.8, 1.0, 1.5, and 2.0 W·cm^−2^. After the CTX@HPDA solution dissolved in PBS was irradiated with a NIR light source (808 nm, 1.0 W·cm^−2^) for 3 min, the NIR light source was turned off for 5 min for natural cooling, and the temperature change of the system was measured every 30 s. The cycle was repeated 5 times to determine the photothermal stability of CTX@HPDA. Using a NIR thermal imaging camera to take photothermal images of CTX@HPDA at different times under NIR radiation.

### 2.6 Animal model establishment and therapeutics

Female C57BL/6 mice (5 weeks old) were purchased from Guangdong Medical Laboratory Animal Center (Foshan, China). Animal work was performed using the protocols approved by the Institutional Animal Care and Use Committee. Female C57BL/6 mice aged 8 weeks were intraperitoneally injected with 0.8 mL of pristane. The model mice were randomly divided into six groups: control, model, CTX, HPDA with 808 nm laser irradiation (1.0 W·cm^−2^), CTX@HPDA, and CTX@HPDA with 808 nm laser irradiation (1 W·cm^−2^). CTX nanoprobes were intraperitoneally injected at a concentration of 1 mg·mL^−1^ and a dosage of 15 mg·kg^−1^ every 4 days. The mice chest was illuminated by NIR laser (808 nm, 1 W·cm^−2^) for 5 min with 1 min interval for each irradiation spot of light exposure after injection of CTX@HPDA or CTX for 2 h. The temperature on mouse chest was monitored using a NIR thermal imaging camera. All mice were treated for 12 days, and their body weights were measured every 2 days.

After 12 days of treatment, the lung areas of different groups of mice were scanned with micro-CT (Aloka, Japan; 80 kV, 40 μA). Scans were done from the level of the upper thoracic inlet to the inferior level of the costophrenic angle. The imaging parameters were as follows: slice thickness, 0.2 mm; voltage, 50 kV; and electricity, 1 mA. Then we performed 3D volume rendering of micro-CT images through RadiAnt DICOM Viewer software. All images were interpreted by a radiologist with 5 years of experience in chest imaging. The radiologist reported the mouse infection spread in percentage based on the judgment of the percentage of infected lung volume. These subjectively assessed values were then collected and performed statistical analyse. The mice were killed at the end of the experiment, and lungs were collected for imaging. The lungs were stained with hematoxylin and eosin (H&E), and structural changes in the lung tissues were observed.

### 2.7 Biosafety assessment of CTX@HPDA

The mice were randomly divided into the experimental and control groups. The experimental group was injected with CTX@HPDA (1 mg·mL^−1^, 100 μL) intraperitoneally, and the control group received an ijection of 100 μL salinesolution. The hearts, livers, spleens, lungs, and kidneys of the mice were collected for histological analysis.

### 2.8 Statistical analysis

Experimental results were shown as mean ± standard deviation. All statistical analyses were performed by Statistical Product and Service Solutions for Windows (SPSS, version 22.0, United States). The significance of differences among groups was assessed using a one-way ANOVA analysis. Statistical significance was indicated by *p < 0.05, **p < 0.01, and ***p < 0.001.

## 3 Results and discussion

### 3.1 Preparation and characterization of CTX@HPDA

**FIGURE 1:**
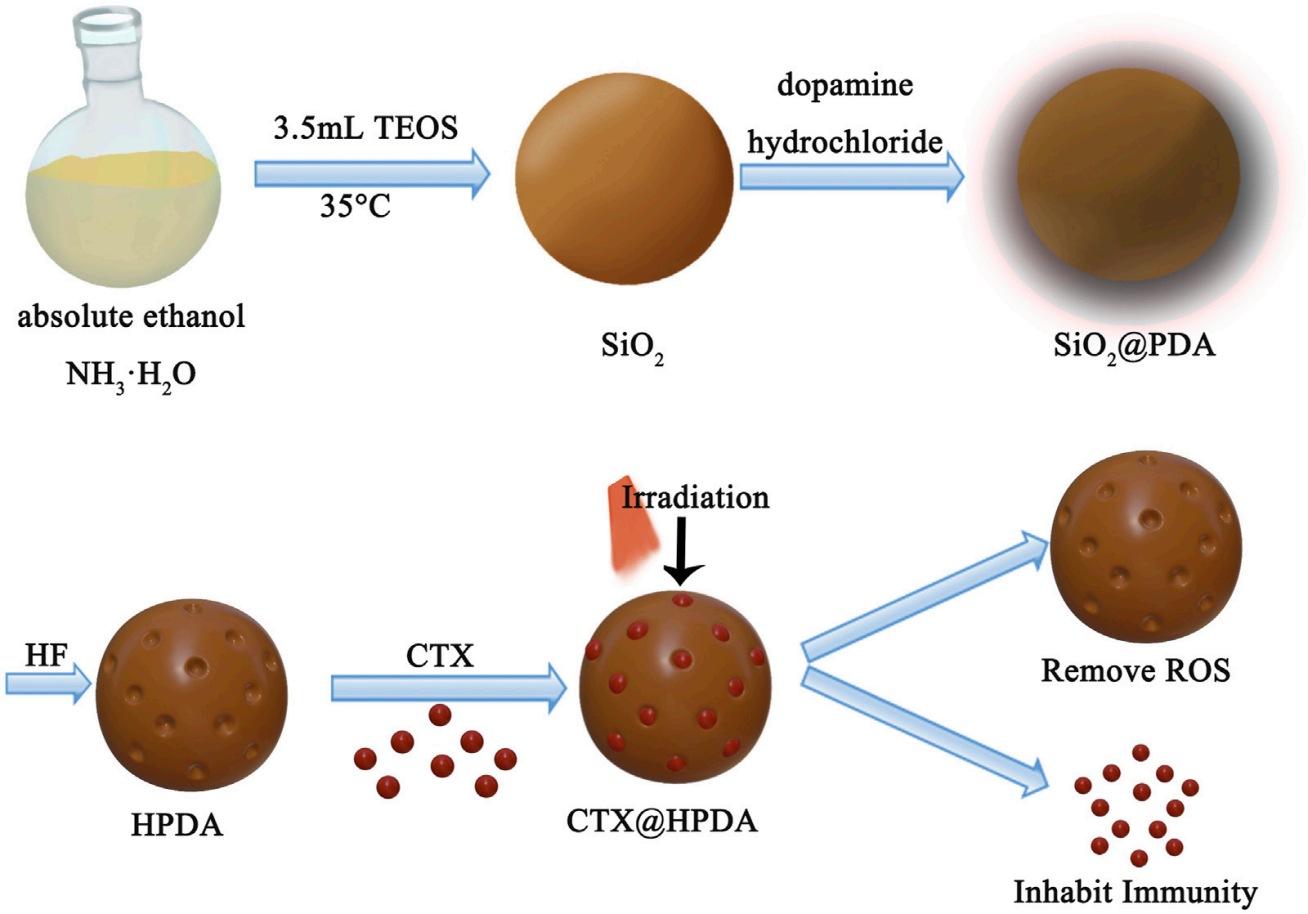
A scheme showing the preparation and application of CTX@HPDA nanoparticles.

**FIGURE 2:**
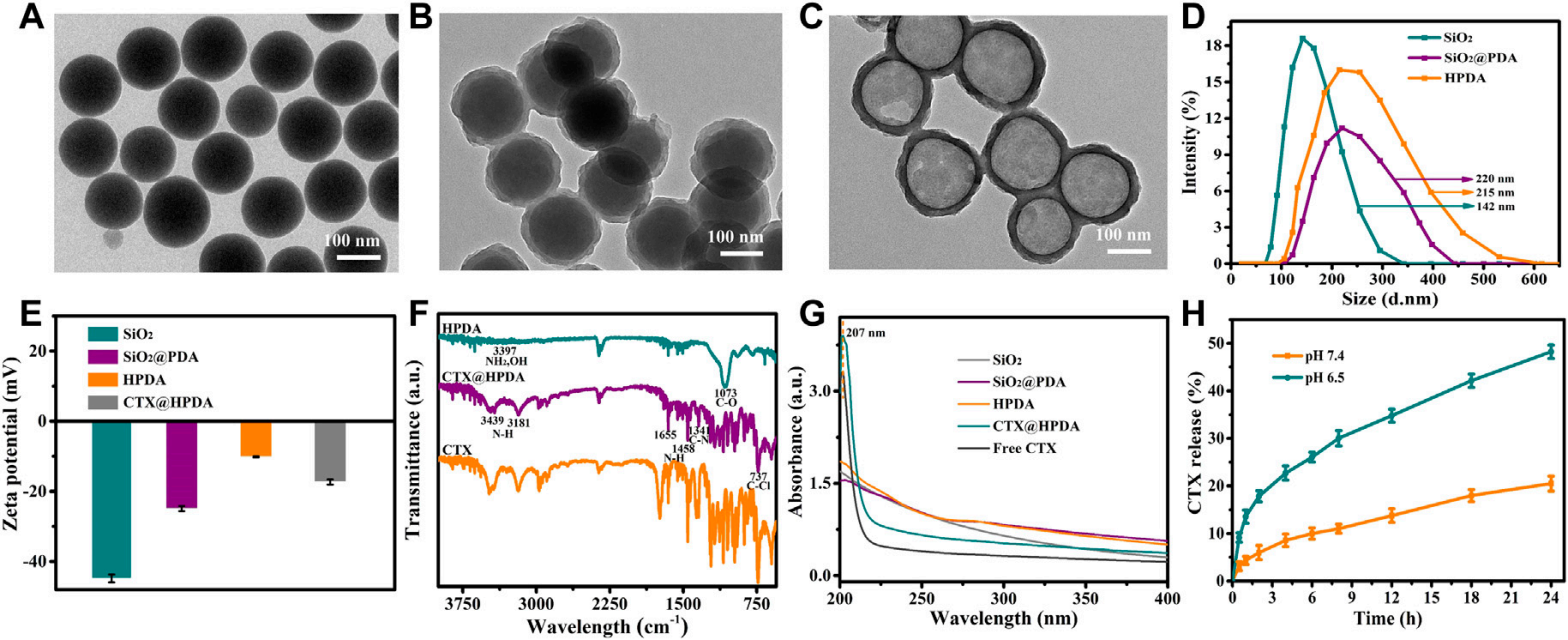
TEM images of **(A)** SiO_2_ NPs, **(B)** SiO_2_@PDA NPs, and **(C)** HPDA NPs. **(D)** DLS of SiO_2_, SiO_2_@PDA, and HPDA NPs. **(E)** Zeta potentials of SiO_2_, SiO_2_@PDA, HPDA, and CTX@HPDA NPs. **(F)** FTIR spectra of HPDA, CTX@HPDA, and free CTX. **(G)** UV-Vis-NIR spectra of the aqueous solutions of SiO_2_, SiO_2_@PDA, HPDA, CTX@HPDA, and free CTX. **(H)** Cumulative CTX release of CTX@HPDA NPs at pH 6.5 and 7.4.

The preparation of CTX@HPDA nanoparticles is shown in Figure 1. SiO_2_ NPs were prepared by typical Stober method. Afterward, PDA was coated on the surface of SiO_2_ by oxidation self-polymerization method to obtain SiO_2_@PDA NPs. Then, HPDA NPs were obtained by HF etching. As demonstrated in Figure 2A, the prepared SiO_2_ NPs were monodispersed with a spherical shape and a diameter of ∼139 nm. Figure 2B shows that SiO_2_@PDA was constructed successfully. HPDA was formed after HF etching (Figure 2C). We also measured the sizes and zeta potentials of SiO_2_, SiO_2_@PDA, and HPDA by DLS (Figures 2D, E), and the average diameters were ∼142, ∼220, and ∼215 nm, respectively, which were marginally larger than the corresponding TEM sizes because DLS showed the average hydrodynamic NP size. Subsequently, the zeta potentials of the NPs were tested (Figure 2E). The zeta potentials of SiO_2_, SiO_2_@PDA, and HPDA were negative because of the existence of silicon hydroxyl groups or phenolic hydroxyl groups on the surface. The zeta potential of CTX@HPDA was decreased to −17.3 mV, which may be due to CTX ionization. The results further proved that CTX@HPDA was successfully prepared.

We compared the FT-IR spectra of HPDA, CTX@HPDA, and free CTX to further verify the encapsulation of CTX. As shown in Figure 2F, the characteristic peaks at 3,397 and 1,073 cm^−1^ are attributed to the NH_2_, O–H, and C–O stretching vibrations of HPDA (Wiercigroch et al., 2017; Nieuwjaer et al., 2018). The characteristic peak at 3,181, 1,341, and 737 cm^−1^ in the spectra of CTX@HPDA are assigned to the N–H, C–N, and C–Cl bond vibrations of CTX. The results indicate that CTX@HPDA was successfully prepared.

Figure 2G shows the UV-Vis-NIR absorption spectra of SiO_2_, SiO_2_@PDA, HPDA, CTX@HPDA, and free CTX. The aqueous solutions of SiO_2_, SiO_2_@PDA and HPDA revealed no obvious absorption peaks. The distinct characteristic absorption peak of CTX at 207 nm appeared in the spectra of CTX@HPDA, indicating that CTX was successfully loaded in CTX@HPDA NPs. In addition, we calculated the loading capacity of CTX. From the UV-Vis-NIR spectrophotometry of HPDA and CTX, the CTX loading capacity of CTX@HPDA NPs was measured to be 23%. These results proved that we successfully prepared CTX@HPDA NPs. Furthermore, the CTX release properties of CTX@HPDA NPs were studied in PBS solutions with pH 6.5 (simulated DAH lesion environment) and pH 7.4 (simulated normal tissue). The results (Figure 2H) showed that 48.2% of CTX was released under pH 6.5 over a 24 h period, which was much higher than that at pH 7.0. The results demonstrated that the CTX released from CTX@HPDA NPs can be controlled by pH.

### 3.2 Photothermal properties of CTX@HPDA NPs

**FIGURE 3:**
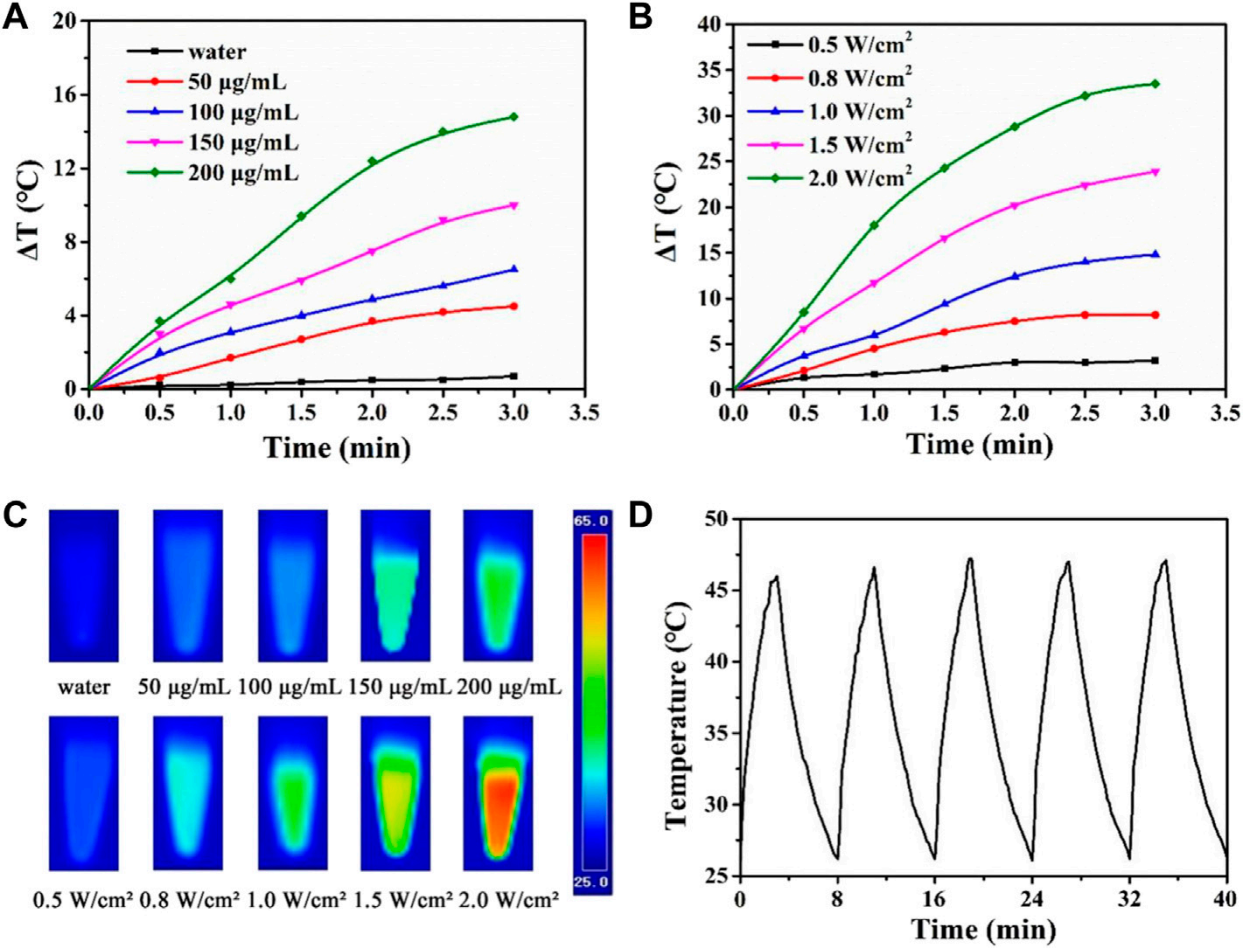
Photothermal properties of CTX@HPDA. **(A)** Photothermal heating curves of CTX@HPDA dispersions at different concentrations under 808 nm laser irradiation (1 W·cm^−2^). **(B)** Photothermal heating curves of CTX@HPDA (200 μg·mL^−1^) dispersions at different power densities. **(C)** Infrared thermal images of different CTX@HPDA solutions upon 3 min of exposure to 808 nm laser irradiation (1 W·cm^−2^, upper row) and 200 μg·mL^−1^ CTX@HPDA under 808 nm laser irradiation with different power densities (bottom row). **(D)** Photothermal stability of CTX@HPDA dispersion (200 μg·mL^−1^) during radiation (1 W·cm^−2^) and natural cooling cycles.

Photothermal therapy is a new technology. Photothermal agents have strong absorption characteristics in the NIR light region and can convert light energy into heat energy. Based on the photothermal properties of PDA nanomaterials, CTX@HPDA photothermal agent can be used for the photothermal treatment for vasculitis. Mild local heat (41°C–43°C) can promote cell proliferation, angiogenesis, causing negligible damage to normal tissue cells in a short time (Zhang et al., 2019). Different CTX@HPDA concentrations (50, 100, 150, and 200 μg·mL^−1^) exhibited a remarkable linear temperature rise under exposure to 808 nm NIR light (1 W·cm^−2^) for 3 min. In particular, the temperature rise was 14.8°C at the concentration of 200 μg·mL^−1^. However, water temperature only increased by 0.7°C, indicating that the absorption of deionized water in the NIR light region is weak (Figure 3A). The temperature changes of CTX@HPDA (200 μg·mL^−1^) within the first 3 min of NIR light irradiation with different power densities (0.5, 0.8, 1.0, 1.5, and 2.0 W·cm^−2^) are shown in Figure 3B. The temperature rise was 33.5°C when the power density was 2.0 W·cm^−2^. The corresponding photothermal images under different conditions are shown in Figure 3C. The results revealed that the temperature of the CTX@HPDA solution increased as the radiation power and concentration increased and had radiation power and concentration dependence. Therefore, temperature can be accurately controlled by adjusting the power density or CTX@HPDA concentration. The CTX@HPDA (200 μg·mL^−1^) solution was irradiated with a NIR light source at 1 W·cm^−2^, and the NIR light source was turned off to cool naturally after 3 min to further investigate the photothermal stability of CTX@HPDA. As shown in Figure 3D, the temperature of CTX@HPDA gradually increased and started to drop naturally after turning off the laser. The temperature of CTX@HPDA NPs under five heating and cooling cycles displayed no substantial attenuation, which indicated good photothermal stability. The results suggested that CTX@HPDA NPs could serve as good photothermal agents for the continuous or repeated irradiation in photothermal therapy.

### 3.3 Therapeutic effect of CTX@HPDA on DAH

**FIGURE 4:**
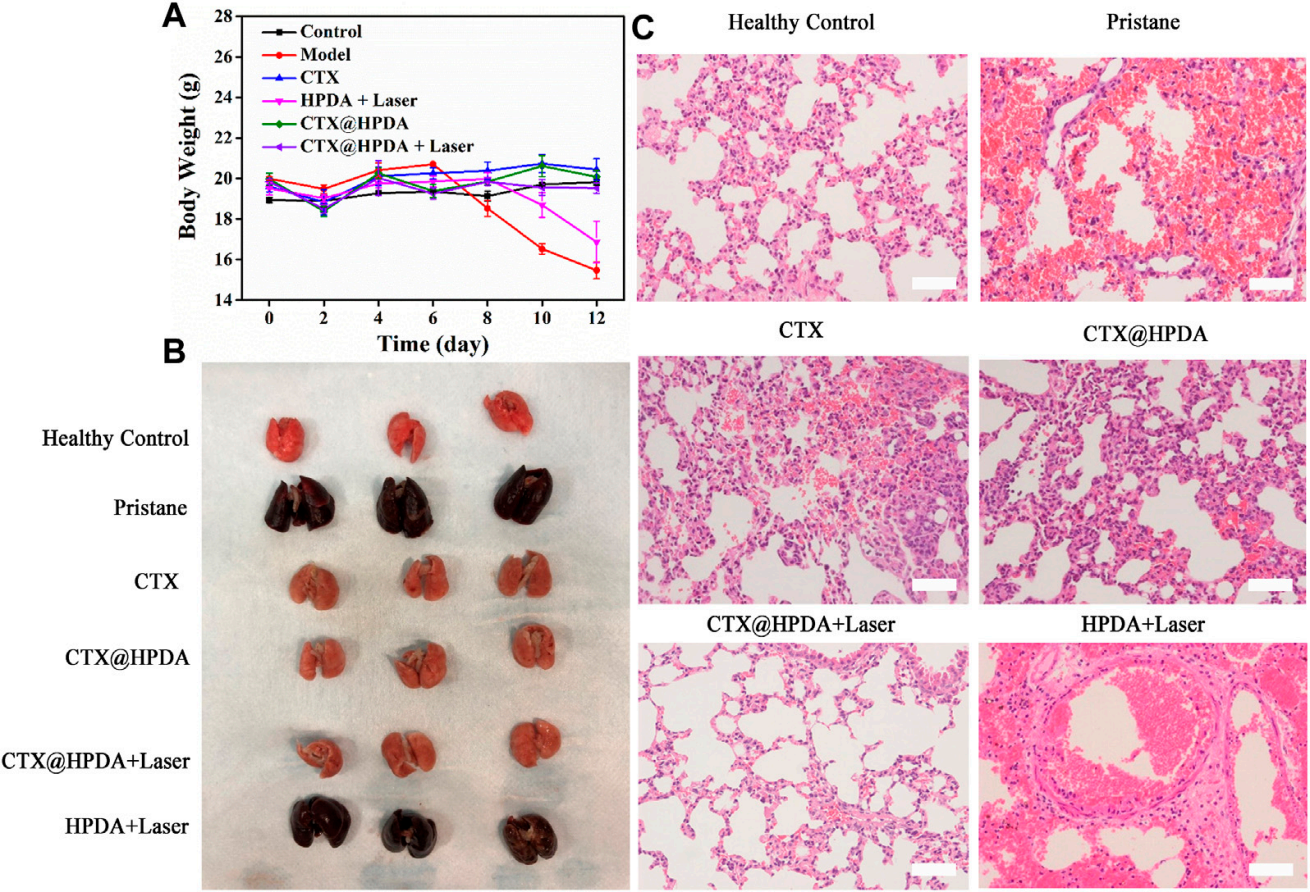
**(A)** Body weights of mice from different groups. Data are presented as mean ± SD. **(B)** Macroscopic view of the bilateral lobes from healthy control mice and mice with pristane-induced diffuse alveolar hemorrhage (DAH) (pristane) with or without different therapy. **(C)** H&E staining images of lung sections (scale bar, 200 µm).

CTX@HPDA showed considerable therapeutic quality in DAH. ROS is one of the main causes of various inflammatory diseases HPDA, as the main material of this nanocomposite, has a powerful role in scavenging free radicals (Liu et al., 2017). Under the action of NIR, HPDA slowly released CTX in the diseased lung tissue, which eliminated the ROS produced in inflammatory reactions. In addition, the acidic environment of SLE also accelerates the release of CTX (Cheng et al., 2017). Body weight changes in the mice were recorded in the entire experiment. The result showed that the body weight of DAH mice (model group) decreased after the sixth day of the experiment. The possible reason for this trend was the rapid progression of DAH after 6 days, which seriously affected the body function of the mice. The weight of the mice in the HPDA + Laser group began to decrease on day 8, indicating that HPDA alone could not prevent the development of the disease. Mice in the control, CTX, CTX@HPDA, and CTX@HPDA + Laser groups, had stable body weights, suggesting that CTX and CTX@HPDA can treat DAH to some extent (Figure 4A).

Gross lung pathology was classified into three degrees of severity according to the percentage of hemorrhage as follows: no hemorrhage (0%), partial hemorrhage (25%–75%), and complete hemorrhage (75%–100%) (Guo et al., 2021). As shown in Figure 4B, the lung tissue in the healthy control group was reddish and had no hemorrhage and tissue swelling (no hemorrhage). However, in the pristane group and HPDA + Laser group, the lung tissue was dark red with massive or diffuse hemorrhage in each lung lobe (complete hemorrhage), and the lung tissue was substantially swollen. In the CTX and CTX@HPDA groups, scattered punctate hemorrhage was observed in some lung lobes (partial hemorrhage), and the swelling of lung tissue was not obvious. The lung tissue of the CTX@HPDA + Laser group was slightly dark and had no hemorrhage or tissue swelling (no hemorrhage). We performed lung pathological examination on the mice after 12 days of treatment for further testing the therapeutic efficacy of CTX@HPDA for DAH. As shown in Figure 4C, H&E staining showed diffuse hemorrhage, destruction of lung tissue structure, and infiltration of inflammatory cells in the lung tissue of the model and PDA + Laser groups. The alveolar hemorrhage and inflammatory infiltration in the lung tissue of mice in each treatment group were considerably reduced, especially in the CTX@HPDA and CTX@HPDA + Laser group, and were close to those in the normal lung tissue. A small amount of alveolar hemorrhage was observed in the lung tissue of the CTX group. These results suggested that CTX@HPDA can ameliorate DAH. In summary, HPDA or CTX alone cannot completely alleviate DAH. Combining the oxygen free radical-scavenging effect of HPDA and the immunosuppressive effect of CTX can alleviate DAH progression of DAH, thus reducing pulmonary hemorrhage and exudation.

### 3.4 Micro-CT image tracking *in vivo*

**FIGURE 5:**
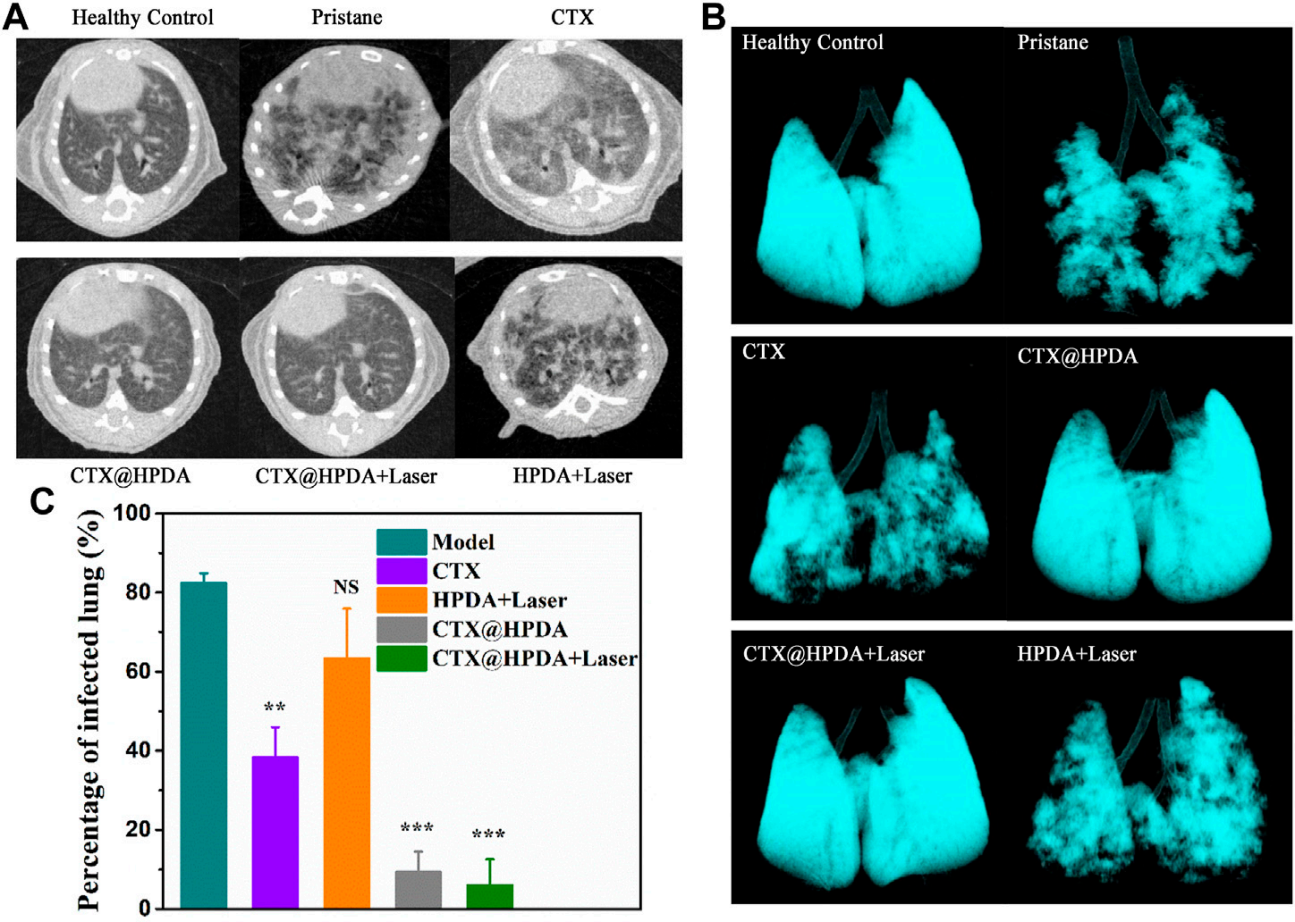
**(A)** Micro-CT images of mice from healthy control mice and mice with pristane-induced diffuse alveolar hemorrhage (DAH) (pristane) with or without different therapy. **(B)** The 3D volume rendering of micro-CT images from different groups. **(C)** Comparison of percentage of infected lung in different groups of mice.

DAH diagnosis in patients without bronchoscopic alveolar lavage and lung biopsy is very difficult. CT, as a non-invasive diagnostic method, can assist in DAH diagnosis (Castañer et al., 2010). Micro-CT examination of small animals is highly sensitive, repeatable, and non-invasive, which is helpful to continuously analyze DAH severity and lung changes in mice after treatment. Figure 5A shows the chest CT scans of mice in the DAH group. The presence of imaging features, such as ground-glass opacity, crazy paving, air space consolidation, reticulation, and bronchial wall thickening, were described. The pulmonary exudation in the various treatment groups improved to varying degrees. The CTX@HPDA + Laser group had the most remarkable therapeutic effect and showed clear bilateral lung fields close to that in the normal lung. Obvious exudation was still found in the PDA + Laser group. The 3D volume rendering of micro-CT images enables clear visualization of the overall lung structure (Figure 5B). Moreover, percentage of infected lung among model and different drug groups were compared (Figure 5C). The results demonstrated that the percentage of infected lung in the CTX@HPDA and CTX@HPDA + Laser group were lower than that in the model group (*p* < 0.001). But The HPDA + Laser group showed only a marginal decrease in the percentage of infected lung tissue. CT results further demonstrated that photothermal therapy combined with immunosuppressive therapy had a remarkbale therapeutic effect on DAH.

### 3.5 Biosafety assessment of CTX@HPDA

**FIGURE 6:**
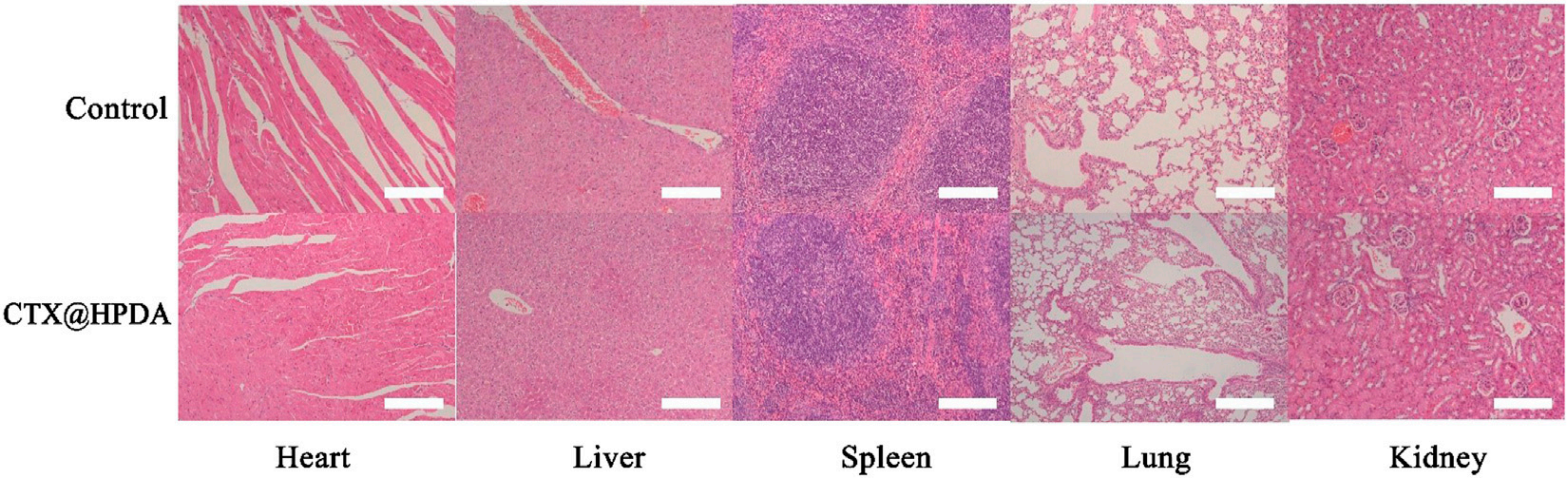
Biosafety and toxicity *in vivo*. Histological toxicity evaluation of CTX@HPDA (scale bar, 200 µm). Organs include the heart, liver, spleen, lungs, and kidney.

Before further studying its clinical application potential, we need to study the biosafety of CTX@HPDA *in vivo*. We stained the important organs (heart, liver, spleen, lung and kidney) of mice with H&E after treatment with CTX@HPDA. The histopathological results showed no obvious abnormality or damage in the important organs of mice (Figure 6), indicating that CTX@HPDA has low toxicity *in vivo*. Therefore, CTX@HPDA may be used for the clinical treatment of DAH.

## 4 Conclusion

In this study, we report a photothermal-triggered nanocarrier for the precise treatment of SLE-DAH. The nanocarrier (CTX@HPDA) consists of HPDA nanomaterials and immunosuppressant CTX and triggers drug release through photothermal conversion and low pH. The release of CTX in the lung was controlled *in vitro* by adjusting the power of NIR light. The vitro experiments demonstrated that CTX@HPDA has good photothermal conversion ability. CTX@HPDA is regarded as safe, with no toxicities observed in mice after receiving the nanocarrier. *In vivo* tests in DAH mouse models confirmed that the nanocarrier could remarkably alleviate DAH progression by combining the immunosuppressive effect of CTX and the free radical-scavenging activities of PDA after 3 weeks of systemic administration. Therefore, CTX@HPDA is a simple and efficient nanocarrier system for DAH therapy. Finally, the full extent of CTX@HPDA in DAH mouse models remains to be explored. We believe that this study may provide new insights into the treatment of other autoimmune diseases, which is the basis for the further studies.

## Data Availability

The original contributions presented in the study are included in the article/supplementary material, further inquiries can be directed to the corresponding authors.
